# HIV-1 Tat enhances purinergic P2Y4 receptor signaling to mediate inflammatory cytokine production and neuronal damage via PI3K/Akt and ERK MAPK pathways

**DOI:** 10.1186/s12974-019-1466-8

**Published:** 2019-04-04

**Authors:** Feng Zhou, Xiaomei Liu, Lin Gao, Xinxin Zhou, Qianwen Cao, Liping Niu, Jing Wang, Dongjiao Zuo, Xiangyang Li, Ying Yang, Minmin Hu, Yinghua Yu, Renxian Tang, Bong Ho Lee, Byoung Wook Choi, Yugang Wang, Yoshihiro Izumiya, Min Xue, Kuiyang Zheng, Dianshuai Gao

**Affiliations:** 1 Jiangsu Key Laboratory of Brain Disease Bioinformation, Research Center for Biochemistry and Molecular Biology, Xuzhou Medical University, Xuzhou, Jiangsu 221004 People’s Republic of China; 2 Jiangsu Key Laboratory of Immunity and Metabolism and Department of Pathogen Biology and Immunology, Xuzhou Medical University, 209 Tongshan Road, Xuzhou, 221004 Jiangsu China; 3 Xuzhou Key Laboratory of Neurobiology, Department of Neurobiology and Anatomy, Xuzhou Medical University, Xuzhou, 221004 Jiangsu China; 4 Department of Chemical and Biological Engineering, Hanbat National University, Dongseodaero 125, Yuseong-gu, Daejeon, 305-719 South Korea; 5 Department of Dermatology, University of California Davis (UC Davis) School of Medicine, Sacramento, CA USA; 6 Department of Physiology, Xuzhou Medical University, Xuzhou, 221004 Jiangsu China

**Keywords:** HIV-associated neurocognitive disorders, Astrocytes, Inflammatory cytokines, Purinergic P2Y4 receptor, Tat

## Abstract

**Background:**

HIV-associated neurocognitive disorders (HANDs) afflict more than half of HIV-1-positive individuals. The transactivator of transcription (Tat) produced by HIV virus elicits inflammatory process and is a major neurotoxic mediator that induce neuron damage during HAND pathogenesis. Activated astrocytes are important cells involved in neuroinflammation and neuronal damage. Purinergic receptors expressed in astrocytes participate in a positive feedback loop in virus-induced neurotoxicity. Here, we investigated that whether P2Y4R, a P2Y receptor subtype, that expressed in astrocyte participates in Tat-induced neuronal death in vitro and in vivo.

**Methods:**

Soluble Tat protein was performed to determine the expression of P2Y4R and proinflammatory cytokines in astrocytes using siRNA technique via real-time PCR, Western blot, and immunofluorescence assays. Cytometric bead array was used to measure proinflammatory cytokine release. The TUNEL staining and MTT cell viability assay were analyzed for HT22 cell apoptosis and viability, and the ApopTag® peroxidase in situ apoptosis detection kit and cresyl violet staining for apoptosis and death of hippocampal neuron in vivo.

**Results:**

We found that Tat challenge increased the expression of P2Y4R in astrocytes. P2Y4R signaling in astrocytes was involved in Tat-induced inflammatory cytokine production via PI3K/Akt- and ERK1/2-dependent pathways. Knockdown of P2Y4R expression significantly reduced inflammatory cytokine production and relieved Tat-mediated neuronal apoptosis in vitro. Furthermore, in vivo challenged with Tat, P2Y4R knockdown mice showed decreased inflammation and neuronal damage, especially in hippocampal CA1 region.

**Conclusions:**

Our data provide novel insights into astrocyte-mediated neuron damage during HIV-1 infection and suggest a potential therapeutic target for HANDs.

**Electronic supplementary material:**

The online version of this article (10.1186/s12974-019-1466-8) contains supplementary material, which is available to authorized users.

## Background

HIV-associated neurocognitive disorders (HAND) include asymptomatic neurocognitive impairment (ANI), mild neurocognitive disorder (MND), and HIV-associated dementia. Although highly active antiretroviral therapy (HAART) reduced viral loading and prolonged life-span of acquired immunodeficiency syndrome (AIDS) patients, the prevalence of HAND appears to be rising up to more than 50% among human immunodeficiency virus type 1 (HIV-1)-positive individuals. HAND is characterized by degenerative damage and loss of neurons and ultimately develops into HIV-associated dementia (HAD) [1–4]. In the central nervous system (CNS), HIV-1 itself and HIV-1-encoded proteins including transactivator of transcription (Tat) enhance altered neuroinflammation and neurotoxicity in astrocytes and microglia, contributing to the neuron damage and loss in the process of HAND [5–8].

Reactive astrocytosis is an important feature in inflammatory environment during the pathology of HAND [5, 9, 10]. Astrocytes, the major glial cell type within the brain [11, 12], represent an important reservoir for the production of various mediators of inflammation, particularly in response to HIV-1 infection [13, 14]. Astrocytes support neuron function through promoting synaptic formation and plasticity, and architecture the formation of the blood-brain barrier (BBB) [10]. Additionally, activated astrocytes are able to interact with the peripheral immune system by recruiting leukocytes and monocytes into the CNS [15]. Meanwhile, astrocyte activation is associated with the pathogenesis of HAND characterized by an increased the production of pro-inflammatory cytokines and chemokines [3, 5]. In response to HIV-1 or Tat, astrocytes elicit chemokines and chemokine production to promote the recruitment of T cells and monocytes into the brain [5]. HIV-infected monocytes and T cells not only infect brain resident cells but also release proinflammatory cytokines such as tumor necrosis factor (TNF) and interleukin-1 beta (IL-1β), which further activates astrocytes [5]. Of important, these activated astrocytes are important contributors to neuroinflammation and release neurotoxic factors such as excitatory amino acids and adenosine 5′-triphosphate (ATP), resulting in neuronal dysfunction and death [16, 17].

Tat, a multifunctional protein of 86-101 aa, is produced from HIV-1-infected cells and releases into the tissues and blood in HIV-1^+^ individuals. Meanwhile, Tat is secreted from resident CNS cells such as microglia and astrocytes into the CSF [18], and increases the production of soluble neurotoxic factors including inflammatory cytokines [5], which involved in the development and progression of HAND in HIV-1^+^ patients [19–24]. Studies showed that Tat induced pro-inflammatory cytokines like interleukin-6 (IL-6), IL-8, and interferon-gamma-inducible 10-Kd protein (IP-10) in astrocytes, which involved nuclear factor-kappa B (NF-κB), AP-1, C/EBPα, and C/EBPγ transcription factors and JAK, PI3K/Akt, and MAPK signaling pathways [25–27]. In addition, some studies reported that Tat induced neurotoxic factors such as excitatory amino acids, ATP, and calcium overload, resulting in neuron death [28, 29]. Therefore, Tat protein treatment in mice can induce key aspects of HAND neurotoxicity [30].

Purinergic P2 receptor family includes P2Y1R, P2Y2R, P2Y4R, P2Y6R, P2Y12R, and P2Y14R that express in CNS astrocytes [31, 32]. Recent data have demonstrated that ATP is recognized to act through purinergic P2 receptors widely expressed in the brain [33], which are involved in glia-glia and glia-neuron communications [34], thus playing important physiological and pathophysiological roles in a variety of biological processes and neurodegenerative diseases [35]. Previous evidence has documented that a non-selective P2 receptor antagonist, PPADS, improves the morphological and functional alterations provoked by the ischemic injury [36]; A P2Y1 agonist, 2-MeSADP, significantly reduces cytotoxic edema and the magnitude of ischemic lesions [37]. Inhibition of P2Y1 receptors in astrocytes results in cytokine/chemokine transcriptional suppression, which involves the NF-kB pathway [38]. Moreover, PPADS confers neuroprotection against glutamate/NMDA toxicity [39]. Recent studies found that purinergic receptor P2Y6 contributes to 1-methyl-4-phenylpyridinium-induced oxidative stress and cell death [40]. PI3K/AKT and ERK MAPK pathways are involved in activating purinergic P2 receptor [41–43]. These results prompt us to determine purinergic P2 receptor signaling in astrocytes and their roles in HAND pathogenesis.

In the present study, we analyzed purinergic P2 receptor expression in astrocytes treated by soluble Tat protein. Our results found that Tat enhanced P2Y4R signaling to mediate inflammatory process that contributed to neuron apoptosis and death via PI3K/AKT- and ERK-dependent pathways. Furthermore, P2Y4R knockdown in mice significantly suppressed inflammatory cytokine production and relieved hippocampal CA1 neuron apoptosis in Tat-treated mice. These data support a role of P2Y4R in regulating neuroinflammation and neuron damage during the process of HAND.

## Methods

### Animals

C57BL/6 mice were purchased from Nanjing University Laboratory Animal Center. Mice were bred and maintained under specific pathogen-free conditions. All experimental procedures described in our study were carried out based on the Provision and General Recommendation of the Chinese Laboratory Association. The protocol was approved by the Institutional Animal Care and Use Committee of Xuzhou Medical University.

### Reagents and antibodies

Full-length recombinant Tat (1-101 aa, ab83353) was from Abcam. ATP (A1852), LY294002 (440202), and PD98059 (P215) were from Sigma-Aldrich. Multiplex magnetic bead-based antibody detection kits (cytokine and chemokine detection kits, Cytometric Bead Array) were purchased from BD Biosciences (560485, 558342). LV3-sh-NC and LV3-sh-mouse P2Y4R were purchased from GenePharma (Shanghai, China). TUNEL staining kit (11684817910) was from Roche. The ApopTag® peroxidase in situ apoptosis detection kit (S7100) was purchased from Chemicon International, Inc. Anti-GFAP antibodies (ab4648 and MAB360) were from Abcam and Millipore. Anti-P2Y4R antibody (APR-006) was from Alomone labs. Anti-p-AKT (Ser473, 4060) and anti-p-ERK1/2 (Thr202/Thr204, 5726) antibodies were from Cell Signaling Biotechnology. Anti-Bcl-2 (sc-509) antibody, anti-Bax (sc-20067) antibody, anti-β-Actin (sc-47778), and anti-Tubulin antibodies were purchased from Santa Cruz Biotechnology. Alexa Fluor® 488 donkey anti-mouse IgG (A21202) and Alexa Fluor® 594 donkey anti-mouse IgG (A21207) antibodies were from Life technologies. The secondary antibodies of goat anti-mouse IgG and goat anti-rabbit IgG were purchased from Santa Cruz Biotechnology.

### Cell culture

Isolation and culture of primary mouse astrocytes from 0- to 1-day-old C57BL/6 mice were established as previously described [44–46]. Briefly, the cerebral cortices freed of meninges were dissected, minced, and digested. The cells were filtrated, transferred to culture flasks pre-coated with 1 mg/ml poly-l-lysine (Sigma-Aldrich), and then cultured DMEM/F12 medium containing 10% fetal bovine serum (FBS). Cells were passed for three passages, and then detected glial fibrillary acidic protein (GFAP, astrocytic marker) expression through immunofluorescence assays (IFA). Finally, at least 95% GAFP^+^ cells were used to research.

Murine hippocampal neuron HT-22 cells and human glioma U261 cells were obtained from the Cell Bank of Chinese Academy of Science (Shanghai, China). HT-22 and U261 cells were cultured in DMEM supplemented with 10% heat-inactivated FBS (fetal bovine serum), 50 U/ml penicillin and streptomycin (Sigma-Aldrich) under a humidified atmosphere containing 5% CO_2_ at 37 °C.

### RNA extraction, reverse transcription, and real-time PCR assay

Total RNA was extracted from astrocytes and hippocampal tissues according to manufactures instructions. Reverse transcription and real-time PCR (qPCR) assay was performed as previously described [47]. The sequences of primers are listed in Additional file 1: Table S1.

### Western blotting analysis

Total protein was extracted from mouse hippocampal tissues and from primary astrocytes as described previously [48]. The expression of protein in samples was normalized by β-actin or Tubulin.

### Immunofluorescence assay

Protein expression was performed by the immunofluorescence assay (IFA) as previously described [49].

### Cytometric bead array

The concentration of inflammatory cytokines was measured using a cytometric bead array (CBA; Multiplex magnetic bead-based antibody detection kits, BD Biosciences, CA, USA) as per manufacturer’s instructions. Conditioned media samples from astrocytes were spun at 160×g for 5 min and collected and stored at − 20 °C. CBA samples were run on a FACSCanto™ II flow cytometer (BD Biosciences, CA, USA). Data were analyzed using the BD FACSDiva software and normalized as described previously [46, 50].

### MTT cell viability assay

Cell viability was examined by MTT assays according to standard methods as previously described [48].

### ATP measurement

ATP levels were measured using the ATPlite luminescence assay system (PerkinElmer, 6016943) according to the manufacturer’s instructions [51].

### Lentivirus and Tat administration in mice

To silence the mouse P2Y4R, shRNA sequences were designed. ShRNA sequences were as followed: sh-negative control, 5′-TTC TCC GAA CGT GTC ACG T-3′; sh-P2Y4R-1, 5′-CCU GUU GCC UCU GAG CUA UTT-3′ and sh-P2Y4R-2, 5′-CCA CUU ACA UGU UCC AUU UTT-3′; sh-P2Y4R-3, 5′-CCT GTT GCC TCT GAG CAT T-3′. ShRNA sequences were cloned into the LV3-eGFP vector (GenePharma), and lentiviruses were packaged, which were named as LV-sh-Ctrl, LV-sh-P2Y4R1/2/3, respectively.

Then, 1 × 10^7^ TU (transduction unit) of LV-sh-Ctrl or LV-sh-P2Y4R were injected into 8-week-old C57BL/6 mice through the tail vein (Additional file 1: Figure S1), followed by Tat protein (1 μg) in 2 μl of PBS was administrated to mice through intracerebroventricular (i.c.v.) infusion site at A/P 1.0 mm, L/M 1.0 mm, and D/V 3.0 mm from the bregma, two times per week for 4 weeks

### Histopathology

Mice were anesthetized and perfusion-fixed with 4% paraformaldehyde. For measuring hippocampal neuron apoptosis, sections (4 μm) from brain tissues were determined by the ApopTag® peroxidase in situ apoptosis detection kit according to the manufacturer’s protocol as previously described [52]. For evaluating hippocampal neuron death, the paraffin-embedded sections (4 μm) from brain tissues were stained with cresyl violet and examined by light microscopy, and the pyramidal cell numbers of surviving in hippocampal CA1/1 mm of length were counted as neuron density [52].

### Statistical analysis

Statistical analysis was performed using SPSS version 18.0 to analyze experimental data. Data presented as mean ± standard error of mean (mean ± S.E.M.). Statistical significance was determined by the two-tailed Student’s *t* test or one-way analysis of variance (ANOVA). *P* < 0.05 was considered significant.

## Results

### HIV-1 Tat promotes P2Y4R expression in primary mouse astrocytes

**Fig. 1:**
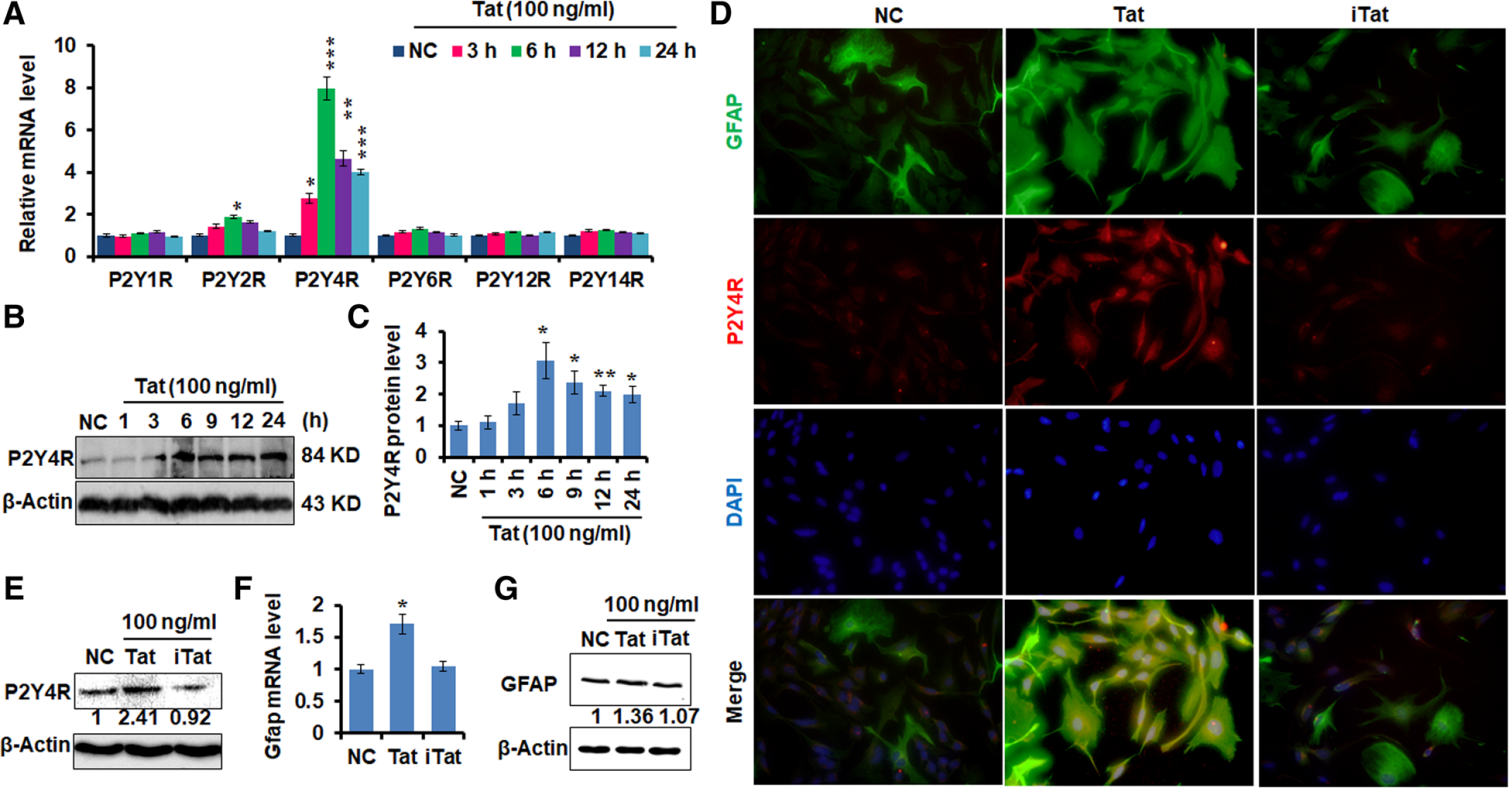
Tat upregulated P2Y4R expression in mouse astrocytes. Primary mouse astrocytes were incubated in a serum-free medium overnight followed by treating with soluble Tat protein for various time points. **a** Representative mRNA levels of P2Y receptors were measured by qPCR assay. These data are from three independent experiments. The relative expression levels of genes were normalized to the expression of mouse *Actin*. *NC* negative control. **b** Representative protein expression of P2Y4R in mouse primary astrocytes was detected by Western blotting assay. **c** Quantification of P2Y4R protein level. Numbers under the bands were the relative intensity of the bands after calculating for loading control using β-actin. The relative value of proteins in NC group was considered as “1”; same for all of following Western blotting Figures. Error bars represent the mean ± S.E.M. These data are from three independent experiments. **P < 0.01 and ***P < 0.001 versus NC group. **d** Immunofluorescent assay for GFAP (green) and P2Y4R (red) in primary mouse astrocytes. Scale bars, 50 μm. *iTat* inactivated Tat, *NC* negative group. **e** Western blotting assay verified protein expression of P2Y4R was in mouse primary astrocytes treated with Tat or iTat for 6 h. The data are from three independent experiments. **f** qPCR assay and **g** Western blotting assay were performed to assess the expression of GFAP mRNA and protein. The relative expression levels of genes were normalized to the expression of mouse *Actin.* Error bars represent the mean ± S.E.M. The data are from three independent experiments. *P < 0.05 versus NC group

To test whether purinergic P2Y receptors involve in Tat-induced astrocyte activation, we first determined P2YR expression after Tat stimulation. Primary mouse astrocytes were treated with 100 ng/ml of soluble full-length recombinant Tat protein in serum-free media; q-PCR assay were performed to measure the transcriptional levels of P2Y1R, P2Y2R, P2Y4R, P2Y6R, P2Y12R, and P2Y14R. As shown in Fig. 1a, the mRNA expression level of P2Y4R was dramatically increased starting at 3 h, peaked at 12 h, and remained high even after 24 h Tat stimulation. Besides P2Y2R mRNA level elevated only at 6 h, the levels of expression of other P2Y receptors were not changed. To determine whether Tat promoted P2Y4R protein expression, immunoblotting and IFA were carried out. Western blotting results indicated that Tat induced P2Y4R expression (Fig. 1b, c), which was correlated with its mRNA expression. Moreover, IFA showed that P2Y4R expression was higher in Tat-treated astrocytes than that of negative control (NC) group (Fig. 1d). To rule out Tat-unrelated effect, we heat-inactivated Tat (iTat) by incubating the recombinant Tat protein in 95 °C for 10 min before adding to primary astrocyte culture. IFA and Western blotting assay indicated that iTat could no longer induce P2Y4R expression (Fig. 1d, e). These results suggest that Tat enhances P2Y4R expression in astrocytes.

Multiple reports have suggested that HIV-1 Tat induces GFAP expression in astrocytes as a marker for tat-induced astrogliosis. As shown in Fig. 1d, Tat appeared to increase GFAP expression. To determine the role of GFAP induction by Tat, q-PCR and immunoblot assay were performed. The soluble Tat enhanced the level of GFAP mRNA and protein (Fig. 1f, g).

### Inhibition of PI3K/Akt and ERK MAPK pathways suppresses Tat-mediated P2Y4R expression and inflammatory response in the primary mouse astrocytes

**Fig. 2:**
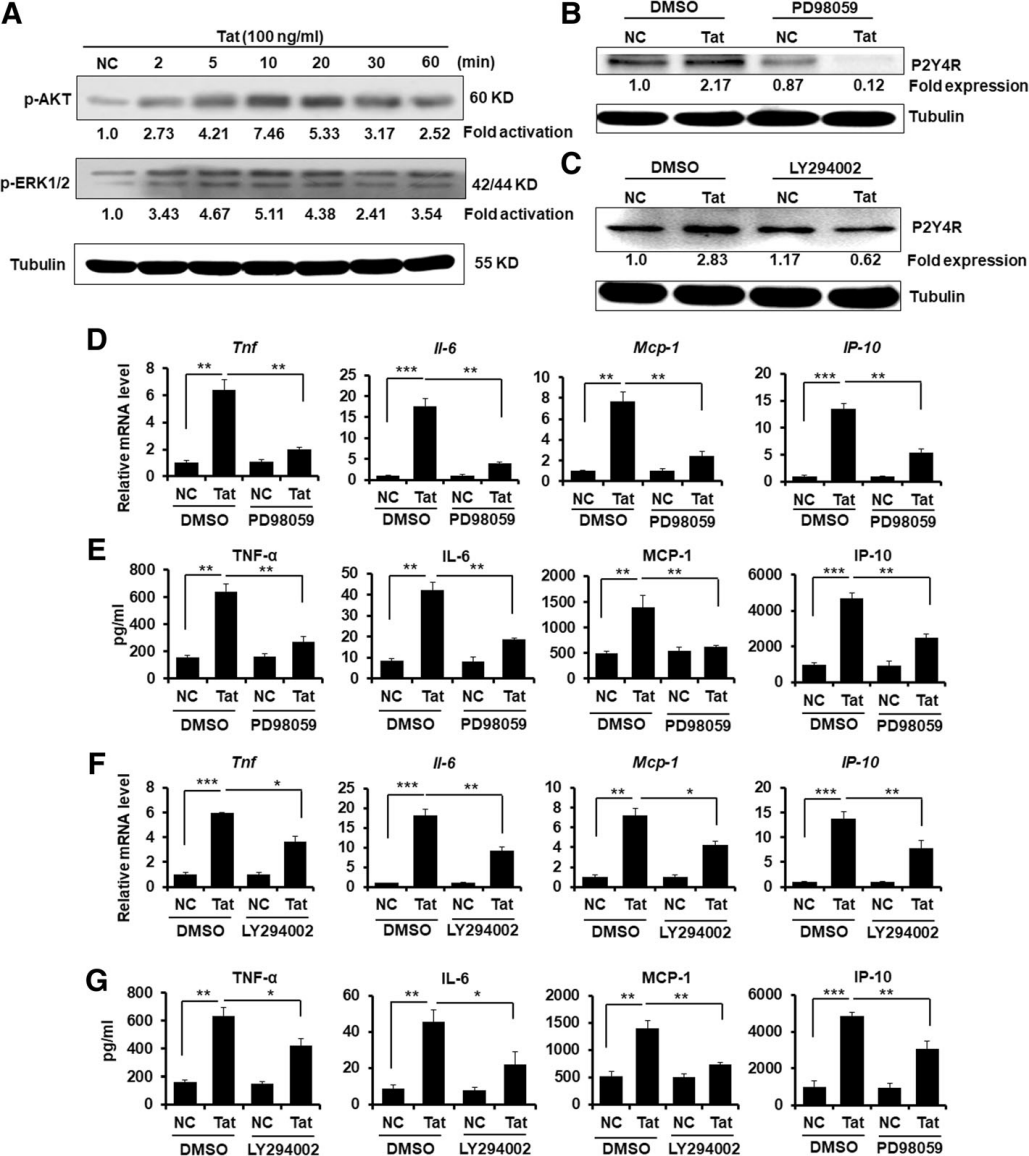
PI3K/Akt and ERK signal pathways were involved in Tat-induced P2Y4R expression and inflammatory response in mouse astrocytes. **a** Primary mouse astrocytes were incubated in a serum-free medium overnight followed by treating with soluble Tat protein for different time point. Representative levels of phosphorylation of Akt (p-Akt) and p-ERK1/2 were displayed by Western blotting assay. These data are from three independent experiments. **b**, **c** Primary mouse astrocytes were incubated in a serum-free medium overnight followed by pretreating with or without PI3K pathway inhibitor LY294002 (10 μM) and ERK1/2 pathway inhibitor PD98059 (50 μM) for 2 h, respectively, prior to soluble Tat protein stimulus for 6 h. Representative levels of P2Y4R protein were determined by Western blotting assay. These data are from three independent experiments. **d**, **e** Primary mouse astrocytes were incubated in a serum-free medium overnight followed by pretreating with or without 50 μM PD98059 for 2 h, prior to soluble Tat protein stimulus for 6 h. **d** Relative mRNA and **e** release levels of TNFα, IL-6, IP-10, and MCP-1were measured by qPCR assay and Cytometric Bead Array, respectively. The data are from three independent experiments. The relative expression levels of genes were normalized to the expression of mouse *Actin.* *P < 0.05, **P < 0.01, and ***P < 0.001. **f**, **g** Primary mouse astrocytes were incubated in a serum-free medium overnight followed by pretreating with or without 10 μM LY294002 for 2 h, prior to soluble Tat protein stimulus for 6 h. **f** Relative mRNA and **g** release levels of TNFα, IL-6, IP-10, and MCP-1were measured by qPCR assay and Cytometric Bead Array, respectively. These data are from three independent experiments. The relative expression levels of genes were normalized to the expression of mouse *Actin.* The data are represented as the mean ± S.E.M. *P < 0.05, **P < 0.01, and ***P < 0.001

To further investigate potential molecular mechanisms by which Tat induces P2Y4R expression, we analyzed the phosphorylation levels of Akt and ERK1/2 in synchronized astrocytes stimulated with Tat protein at different time point. Phosphorylated Akt (p-Akt) was increased by 2.73 times, 4.21 times, 7.46 times, 5.33 times, 3.17 times, and 2.52 times in astrocytes treated with Tat at 2 min, 5 min, 10 min, 20 min, and 30 min, respectively (Fig. 2a). Similarly, the phosphorylation level of ERK1/2 (p-ERK1/2) was risen by 3.43 times, 4.67 times, 5.11 times, 4.38 times, 2.41 times, and 3.54 times in astrocytes treated with Tat at 2 min, 5 min, 10 min, 20 min, and 30 min, respectively (Fig. 2a). The data indicate that Tat activates Akt and ERK1/2 signal pathways.

Then, we assessed whether Akt and ERK1/2 signal pathways were essential in P2Y4R expression induction by Tat. The PI3K/Akt pathway inhibitor LY294002 and ERK1/2 inhibitor PD98059 were applied to primary mouse astrocytes, followed by Tat. Inhibition of both PI3K/Akt and ERK1/2 pathways reduced significantly P2Y4R protein expression (Fig. 2b, c). Thus, Tat triggers PI3K/Akt and ERK1/2 signal pathways to promote P2Y4R expression in astrocytes.

To investigate whether PI3K/Akt and ERK MAPK signals is also required for inflammatory cytokine production during Tat challenges, the levels of TNFα, IL-6, IP-10, and monocyte chemoattractant protein-1 (MCP-1) were monitored. Both PD98059 and LY294002 dramatically reduced mRNA level of *Tnfα*, *Il*-*6*, *IP*-*10*, and *Mcp*-*1* as determined by qPCR in in primary mouse astrocytes stimulated by Tat (Fig. 2d, f). The protein levels of TNFα, IL-6, IP-10, and MCP-1 were also significantly diminished in astrocytes after pretreated by PD98059 and LY294002 (Fig. 2e, g).

### P2Y4R signaling regulates inflammatory response in mouse astrocytes by Tat

**Fig. 3:**
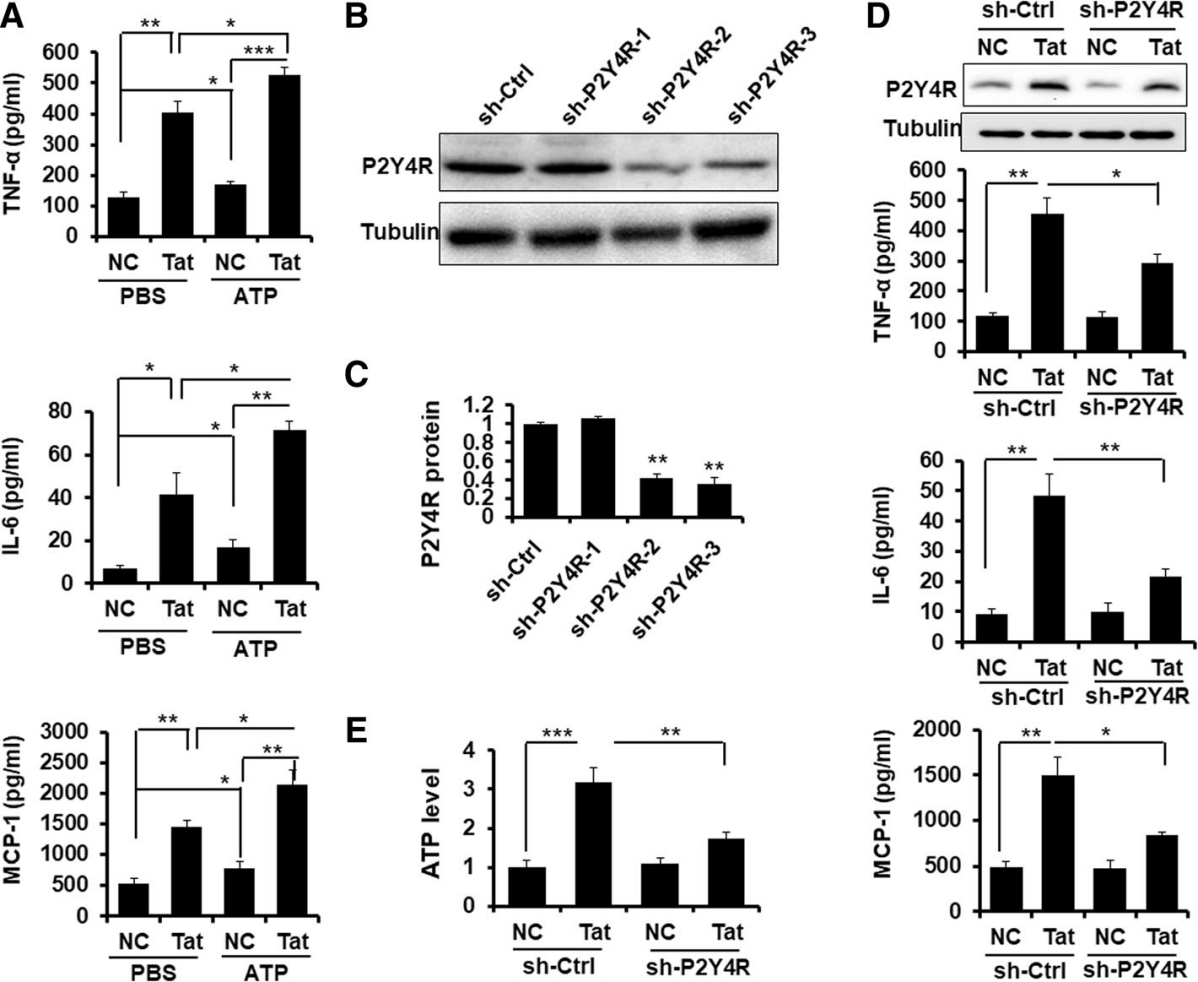
P2Y4R signaling regulates inflammatory response and ATP production in mouse astrocytes by Tat. **a** Primary mouse astrocytes were incubated in a serum-free medium overnight followed by pretreating with or without 100 μM ATP for 2 h, prior to soluble Tat protein stimulus for 6 h. Relative release levels of TNFα, IL-6, and MCP-1were determined by cytometric bead array. These data are from three independent experiments. Error bars represent the mean ± S.E.M. *P < 0.05, **P < 0.01, and ***P < 0.001. **b**, **c** Determination of sh-RNA knockdown of P2Y4R were performed by Western blotting assay in primary mouse astrocytes transduced with sh-Ctrl and sh-P2Y4R1/2/3 for 72 h, respectively. **P < 0.01 versus NC. **d** P2Y4R protein was measured by immunoblotting assay, and relative protein levels of TNFα, IL-6, and MCP-1 were determined by cytometric bead array (*n* = 3). The data are represented as the mean ± S.E.M. *P < 0.05 and **P < 0.01. **e** Relative ATP levels were analyzed by the ATPlite luminescence assay system. The data are from three independent experiments. The data are represented as the mean ± S.E.M. **P < 0.05 and ***P < 0.01

To ascertain whether P2Y4R signal was involved in regulating Tat-induced inflammatory responses, P2Y 4R agonist ATP was applied to primary mouse astrocytes together with or without soluble Tat protein. As shown in Fig. 3a, ATP significantly enhanced the effect of Tat-induced TNFα, IL-6, and MCP-1 expression. To further demonstrate the role P2Y4R in Tat-mediated inflammation, P2Y4R expression was knockdown by shRNA. Sh-P2Y4R-3 significantly suppressed P2Y4R expression (Fig. 3b, c). We then treated the primary mouse astrocytes that had transduced by sh-P2Y4R-3 or sh-Ctrl with Tat for 24 h; the secretion of TNFα, IL-6, and MCP-1 was measured by Cytometric Bead Array. Imminoblot assay confirmed that P2Y4R knockdown inhibited Tat-induced P2Y4R protein, and importantly P2Y4R knockdown decreased the release of TNFα, IL-6, and MCP-1 significantly (Fig. 3d). Overall, these data confirm that P2Y4R signaling participates in Tat-induced proinflammatory cytokine production.

### P2Y4R inhibition decreases the release of ATP in mouse astrocytes by Tat

To determine how Tat engages P2YR signals, ATP production were measured using the ATPlite luminescence assay system. The results indicated that ATP release was dramatically increased in astrocytes treated with Tat, and interestingly, it also significantly reduced in P2Y4R-3-knockdowned cells (Fig. 3e).

### Tat upregulates P2Y4R signaling to mediate inflammatory process and ATP release in human glioma cells via PI3K/Akt and ERK pathways

**Fig. 4:**
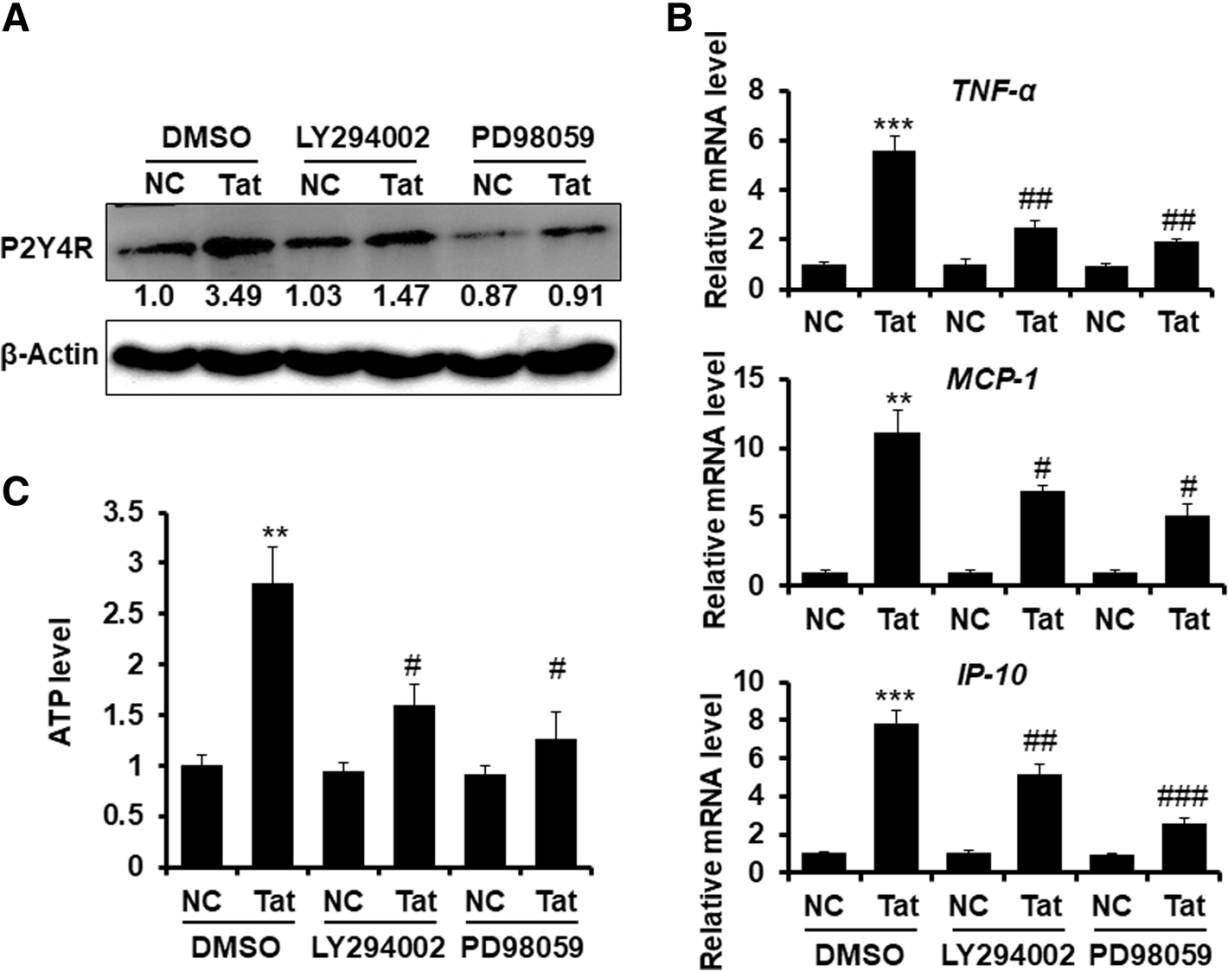
Tat upregulates P2Y4R signaling to produce inflammatory cytokines and ATP release in human glioma cells via PI3K/AKT and ERK pathways. Human glioma U251 cells were pretreated with or without PD98059 and LY294002 for 2 h, followed by soluble Tat stimulus for 6 h. **a** PRY4R protein expression was determined by Western blotting assay. **b** The mRNA levels of *TNFα*, *IL*-*6*, *MCP*-*1* were detected using qPCR. The relative expression levels of genes were normalized to the expression of human Actin. **c** ATP was detected using ATPlite luminescence assay system. The Data are from three independent experiments. The data are represented as the mean ± S.E.M. **P < 0.05 and ***P < 0.01 versus NC with DMSO; ^#^*P* < 0.05, ^##^*P* < 0.01 and ^###^*P* < 0.001 versus Tat with DMSO

To determine whether the Tat-induced P2Y4R pathway also works in human astrocyte, human glioma U251 cells were pretreated with or without PD98059 and LY294002 for 2 h, followed by soluble Tat stimulus for another 6 h, and PRY4R protein expression was then determined by immunoblot assay; the mRNA levels of *TNFα*, *IL*-*6*, *MCP*-*1*, and ATP were detected by qPCR and ATPlite luminescence assay, respectively. Western blotting results indicated that P2Y4R expression was significantly enhanced by Tat, and was reduced by PD98059 or LY294002 (Fig. 4a). Tat-induced *TNFα*, *IL*-*6*, and *MCP*-*1* mRNA expression were dramatically suppressed by PD98059 and LY294002 (Fig. 4b). ATPlite luminescence assay showed that ATP levels were increased by Tat, and was decreased upon treatment with PD98059 or LY294002 (Fig. 4c). These data indicate that soluble Tat enhances P2Y4R signaling in human astrocytes via PI3K/Akt and ERK pathways.

### P2Y4R knockdown displays neuroprotective effect against Tat

**Fig. 5:**
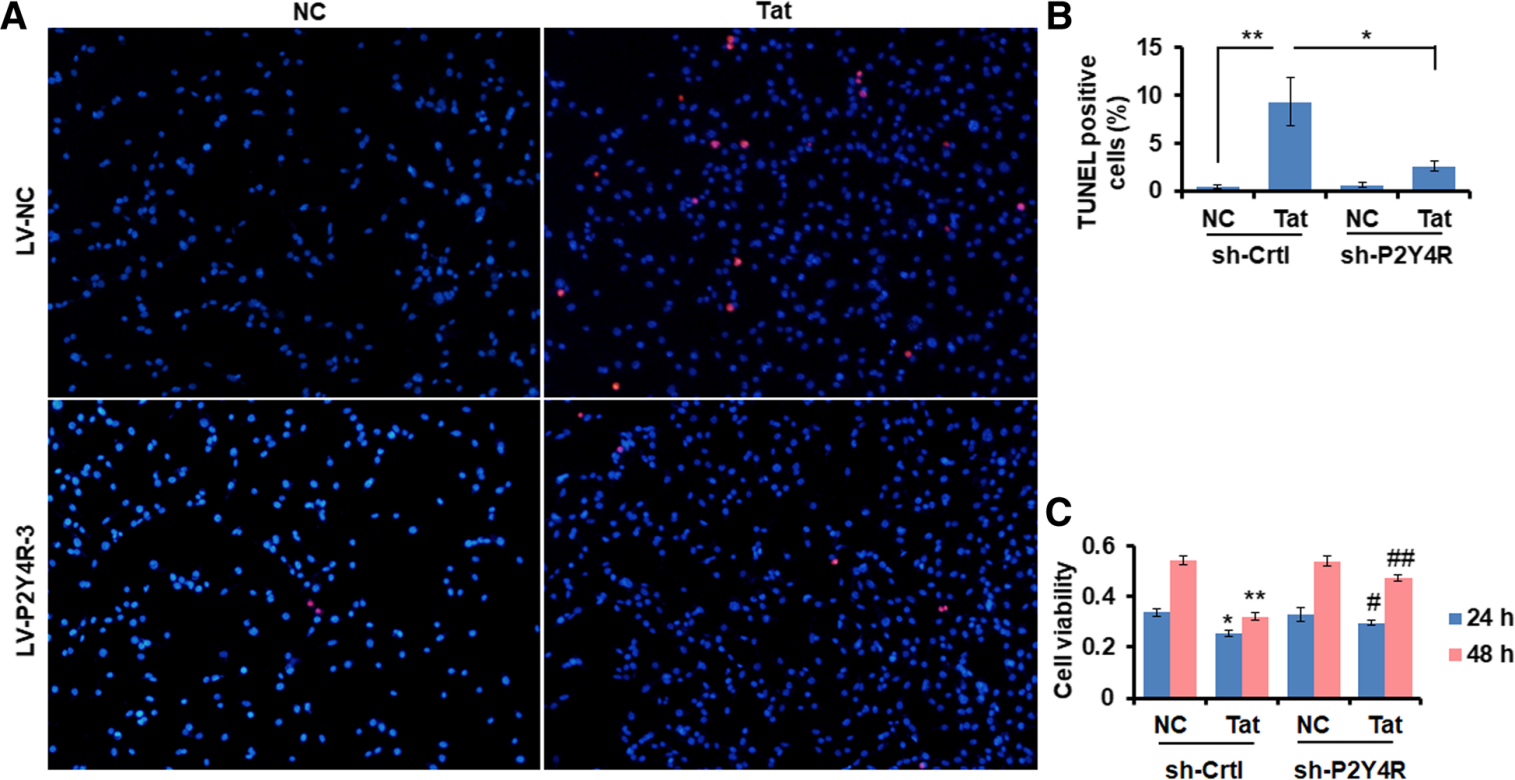
P2Y4R knockdown signaling displays neuroprotective effects against Tat. a Primary mouse astrocytes were transduced with sh-Ctrl and sh-P2Y4R3 for 48 h, followed by Tat stimulus for 24 h. Cell supernatants were collected as astrocyte-derived conditioned media (ACM) and added into HT-22 cells to incubate for 24 h. Representative apoptotic cells were determined by TUNEL staining in HT-22 cells through incubating in ACM for 24 h. Red color as apoptosis positive; blue color of DAPI as nucleus location. **b** Quantification of apoptosis positive cells **a** was calculated through answering for percent of total DAPI positive cells. The data are from three independent experiments. The data are represented as the mean ± S.E.M. *P < 0.05 and **P < 0.01. **c** Relative cell viability was analyzed by MTT assay in HT-22 cells treated with CAM for 24 h and 48 h (*n* = 5). The experiment was independently repeated three times. The data are represented as the mean ± S.E.M. *P < 0.05 and **P < 0.01 versus NC with sh-Ctrl; ^#^*P* < 0.05 and ^##^*P* < 0.01 versus Tat with sh-P2Y4R3

Previous data have documented that P2 receptors are associated with the growth and survival of neurons in the CNS [53]. To explore whether P2Y4R signal protected neuron injury against Tat, primary mouse astrocytes were transduced by LV-sh-P2Y4R-3 or LV-sh-Ctrl for 72 h, followed by Tat stimulus for 24 h, and then cell culture supernatants were collected as astrocyte-derived conditioned media (ACM). The ACM was applied to HT-22 cell, an immortalized mouse hippocampal cell. The apoptotic cell death and cell survival were determined by TUNEL staining and MTT cell viability assay, respectively. TUNEL staining showed that a large number of apoptotic cells were observed in LV-sh-Ctrl-treated group, but which was reversed in LV-sh-P2Y4R-treated group (Fig. 5a, b). Cell viability was decreased in the ACM derived from LV-sh-Ctrl-transduced astrocytes, which was rescued in the ACM from LV-sh-P2Y4R-tranduced astrocytes (Fig. 5c).

### Knockdown of P2Y4R represses inflammation and neuron death of hippocampal CA1 region in Tat-treated mice

**Fig. 6:**
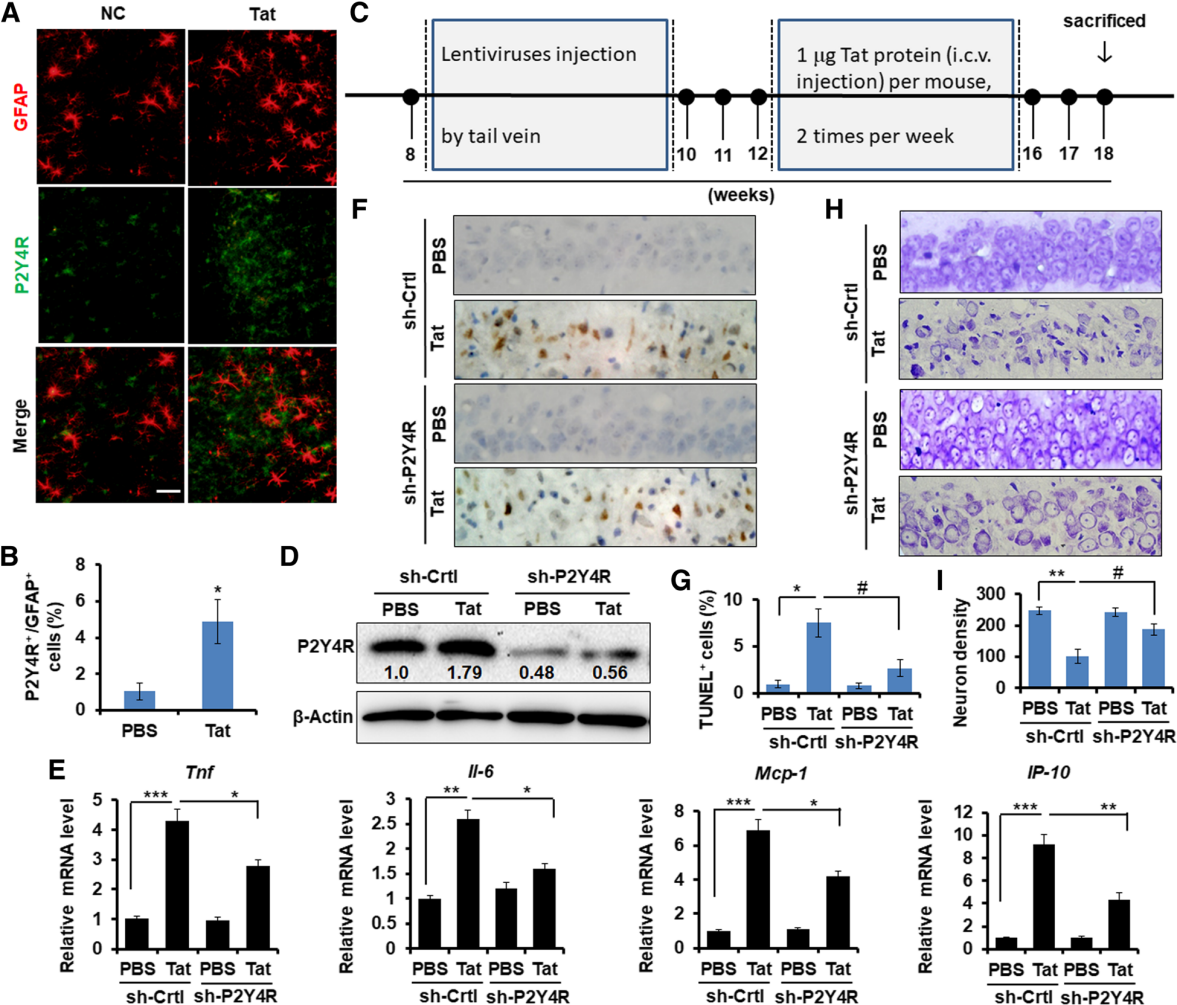
Knockdown of P2Y4R represses inflammation and neuron death of hippocampal CA region in Tat-injected mice. **a** IFA was performed to test GFAP and P2Y4R protein levels in the brain tissue from Tat-treated mice. **b** Calculating the percent of P2YR4 expression in GFAP positive cells. The data are represented as the mean ± S.E.M. *P < 0.05. **c** Schematic diagram of experimental design in mice. Mice were subjected to LV-sh-Ctrl and LV-sh-P2Y4R3 for 14 days before Tat injection, respectively (*n* = 12 mice/group). **d** Representative P2Y4R protein expression was analyzed by Western blotting in hippocampal tissues (*n* = 6). **e** Relative mRNA expression of TNFα, IL-6, MCP-1, and IP-10 was measured using qPCR assay (*n* = 6). The relative expression levels of genes were normalized to the expression of mouse *Actin.* The data are represented as the mean ± S.E.M. *P < 0.05, **P < 0.01 and ***P < 0.001. **f** Representative neuronal apoptosis in hippocampal CA1 subfield photomicrographs was TUNEL staining and counterstaining with methyl green (*n* = 5). Original magnification, × 400. **g** Quantification of the percent of apoptosis positive neurons. Data were obtained from five independent animals and represented as the mean ± S.E.M. *P < 0.05 versus NC with LV-sh-Ctrl; ^#^*P* < 0.05 versus Tat with LV-sh-P2Y4R3. **h** Representative cresyl violet-stained sections of the hippocampal tissues. Original magnification, × 400. **i** Quantification of hippocampal CA1 neuron density. Data were obtained from five independent animals and represented as the mean ± S.E.M. **P < 0.01 versus NC with LV-sh-Ctrl; ^#^*P* < 0.05 versus Tat with LV-sh-P2Y4R3

To further verify the role of P2Y4R in Tat-induced inflammatory process and neuron injury in vivo, IFA was performed to detect whether Tat induce P2Y4R protein level in astrocytes in the brain. As shown in Fig. 6a, b, Tat upregulated the expression of P2Y4R protein in astrocytes. Then, we assessed the effect of P2Y4R blockade by systemic administration of LV-sh-P2Y4R and LV-sh-Ctrl on mice (Fig. 6c). As shown in Fig. 6d, Tat increased P2Y4R protein expression in hippocampal region, but it was suppressed by LV-sh-P2Y4R. The mRNA levels of *Tnfα*, *Il*-*6*, *IP*-*10*, and *Mcp*-*1* were increased in the hippocampal tissues from Tat-injected mice, and were decreased by LV-sh-P2Y4R compared with LV-sh-Ctrl (Fig. 6e). Apoptotic cells of hippocampal neuron were measured by TUNEL staining and cresyl violet staining, respectively. As shown in Fig. 6f, g, compared with the PBS group, numbers of TUNEL-positive cells were significantly increased in the Tat-treated group. Administration of LV-sh-P2Y4R before Tat injection significantly reduced TUNEL-positive cells, comparing with the LV-sh-Ctrl group. Cresyl violet staining displayed that normal CA1 neuronal cells showed round and palely stained nuclei, whereas Tat-induced dead cells showed pyknotic nuclei (Fig. 6h). Administration of LV-sh-P2Y4R significantly decreased neuronal degeneration, compared with LV-sh-Ctrl group (Fig. 6h). Meantime, the neuronal density was dramatically decreased in Tat-treated mice, compared to PBS group; which was increased in LV-sh-P2Y4Rgroup, comparing with LV-sh-Ctrl group (Fig. 6i). Taken together, these results represent that inhibition of P2Y4R protects neuron death against Tat.

## Discussion

**Fig. 7:**
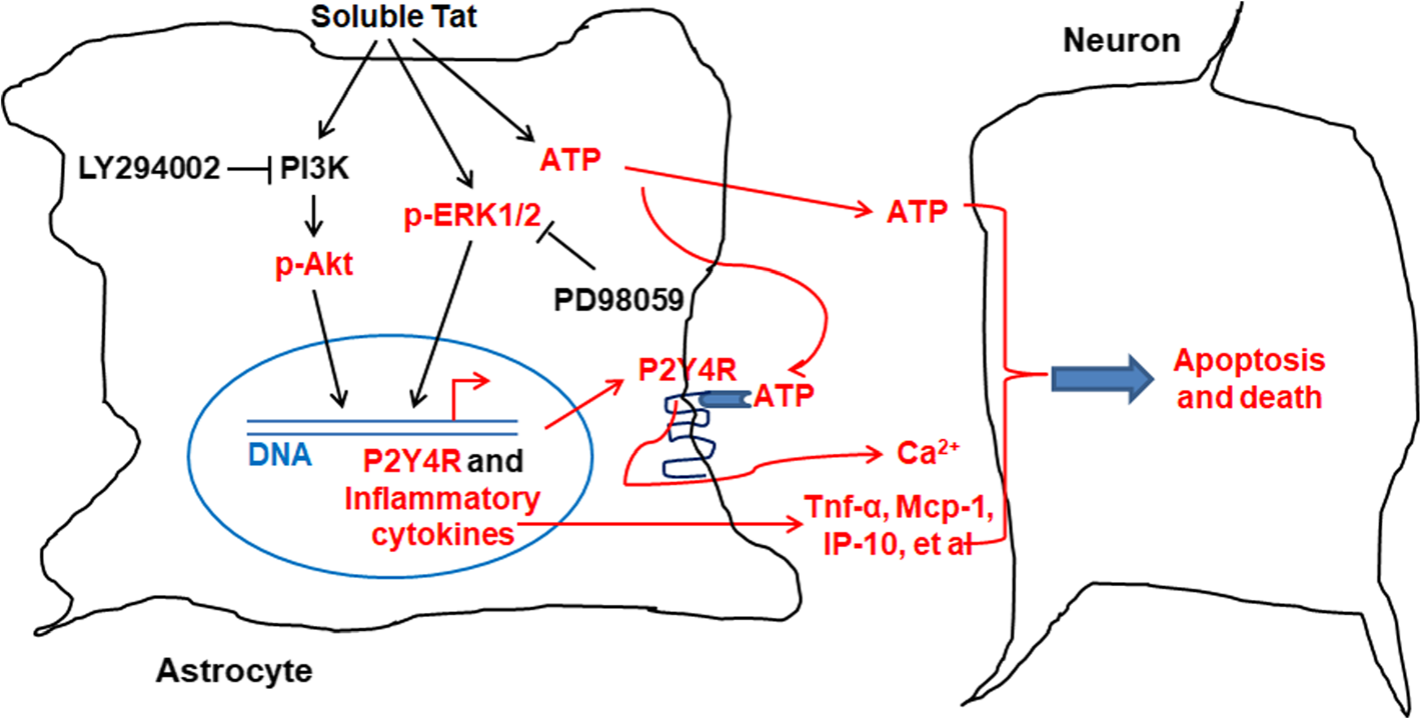
Schematic diagram of P2Y4R signal activated by Tat via PI3K/Akt and ERK pathways in astrocyte-mediated neuron death. Under soluble Tat stimulation, P2Y4R are upregulated in astrocytes via PI3K/Akt and ERK pathways, which increases inflammatory process and ATP release to damage neuron, thus exacerbating the HAND process

A large body of evidence has demonstrated that purinergic P2 receptors play an important role in neuroinflammatory diseases such as Alzheimer’s and Parkinson’s diseases [53, 54]. Growing evidence shows that that P2X7 receptor activation induces large-scale ATP release. ATP acting via P2X7 receptor is the second signal to the inflammasome activation, inducing both maturation and release of pro-inflammatory cytokines, such as IL-1β and IL-18, and the production of reactive nitrogen and oxygen species. Furthermore, the P2X7 receptor is involved in caspases activation, as well as in apoptosis induction [55]. Previous studies reported that Tat enhanced P2X7R expression to mediate neuroinflammation and neuronal damage [56]. However, few studies reported purinergic P2Y receptors on regulating inflammatory process and neuron injury in HAND. In the present study, we aimed to explore the role of purinergic P2Y receptors in regulating astrocytes functions and HAND pathogenesis. Here, we illustrated the effects of P2Y4R on the production of inflammatory cytokines in astrocytes with Tat treatment and neuron death (Fig. 7). Tat augmented P2Y4R expression through activated PI3K/Akt and ERK MAPK pathways to promote production of inflammatory cytokines and ATP. Notably, P2Y4R knockdown reduced inflammation and alleviated hippocampal neuron impairment in Tat-injected mice.

Numerous studies have determined that Tat is a vital neurotoxic mediator [30, 57]. Previous studies exhibited that soluble Tat protein triggers the production of proinflammatory chemokines like IL-8, IP-10, and MCP-1 [8, 25, 56, 57], as well as cytokines including IL-1β, IL-6, and TNF-α in astrocytes [26, 56]. Tat leads to endoplasmic reticulum (ER) stress in astrocytes, which in turn contributes to astrocyte-mediated Tat neurotoxicity [58]. In addition, Tat regulates excitatory amino acids and ATP, resulting in neuron cell death [28, 29]. However, the mechanism by which Tat promotes the production of inflammatory cytokines and ATP in astrocytes and HAND pathogenesis was not completely understood.

PI3K/Akt and ERK MAPK pathways play vital roles in cell growth and differentiation, metabolism, inflammation, and neurodegenerative diseases. In CNS, PI3K/Akt and ERK pathways are activated by ATP or UTP to regulate P2Y4R expression in astrocytes [41, 43]. Furthermore, activation of P2Y4R signaling can promote proinflammatory cytokines production and ATP release [59], in turn triggers calcium overload to exacerbate neuron apoptosis and dysfunction [31, 32, 60]. Here, we demonstrated that the expression of P2Y4R was significantly increased in astrocytes treated with soluble Tat, and suppressed by inhibition of PI3K/Akt and ERK pathways. Meanwhile, Tat increased ATP level via PI3K/Akt and ERK pathways. Of importance, extracellular ATP enhanced Tat-induced production of inflammatory cytokines and chemokines, indicating ATP promoting the productions of inflammatory cytokines through upregulating PI3K/Akt and ERK signals in astrocytes. We also found that reduction of P2Y4R expression by LV-shRNA significantly downregulated inflammatory cytokine expression in hippocampal tissues from Tat-injected mice. It was shown that modulating P2Y4R expression and PI3K/Akt and ERK signal activation would be a potential target for drug development aimed to control neuroinflammation and several types of inflammatory diseases [25, 38, 41, 59].

Inflammation of the CNS is a typical feature of neurological disorders that are characterized by activation of glial cells. In present study, we evaluated that 100 ng/ml of soluble Tat protein significantly triggered inflammatory cytokine production in astrocytes, and administration of 1 μg Tat protein through i.c.v. infusion elicited inflammatory response in mice, which was decreased by P2Y4R knockdown. Notably, P2Y4R knockdown attenuated neuronal apoptosis and death through activated astrocytes by Tat in vitro, protected neuronal apoptosis against Tat, and restored hippocampal CA1 neuron density in vivo.

## Conclusion

Taken together, our data demonstrated that the upregulation of P2Y4R signal both in mice and in activated human astrocytes by Tat regulates the production of inflammatory cytokines and chemokines as well as ATP in astrocytes and in mice via triggering the PI3K/Akt and ERK pathways, which is responsible for HAND process. Thus, our findings on the underlying mechanisms that regulate astrocyte function and the HAND pathophysiological process broaden horizons in understanding of HAND pathophysiology and potential therapeutic strategies.

## Additional file

Additional file 1: **Figure S1.** Distribution of GFP in CNS of mice infected by lentivirus. The lentivirus suspension of LV-sh-P2Y4R was injected into mice through the tail vein for 14 days, and then mice were sacrificed and frozen sections (15 μm) from brain tissues. GFP expression was detected under fluorescence microscope (*n* = 3, original amplification, × 40). **Table S1.** The list of primer sequences for qPCR assay. (ZIP 263 kb)

## Abbreviations

AIDS: Acquired immunodeficiency syndrome
AKT: Protein kinase B
BBB: Blood-brain barrier
CNS: The central nervous system
ERK1/2: Extracellular signal-regulated kinases 1 and 2
GFAP: Glial fibrillary acidic protein
HAART: Highly active antiretroviral therapy
HAD: HIV-associated dementia
HAND: HIV-associated neurocognitive disorder
HIV-1: Human immunodeficiency virus type 1
IL-1β: Interleukin-1 beta
IL-6: Interleukin-6
IP-10: Interferon-gamma-inducible 10-Kd protein
MCP-1: Monocyte chemoattractant protein-1
NF-κB: Nuclear factor-kappa B
P2Y4R: Purinergic P2Y4 receptor
PI3K: Phosphoinosmde-3-kinase
Tat: Transactivator of transcription
TNF: Tumor necrosis factor
