# Supplementary material for: HIV-1 Tat enhances purinergic P2Y4 receptor signaling to mediate inflammatory cytokine production and neuronal damage via PI3K/Akt and ERK MAPK pathways
Source: J Neuroinflammation. 2019 Apr 4;16:71. doi: 10.1186/s12974-019-1466-8 (PMC6449963; doi:10.1186/s12974-019-1466-8)
Supplement: Supplementary file 1 — Figure S1. Distribution of GFP in CNS of mice infected by lentivirus. The lentivirus suspension of LV-sh-P2Y4R was injected into mice through the tail vein for 14 days, and then mice were sacrificed and frozen sections (15 μm) from brain tissues. GFP expression was detected under fluorescence microscope (n = 3, original amplification, × 40). Table S1. The list of primer sequences for qPCR assay. (ZIP 263 kb) [file 12974_2019_1466_MOESM1_ESM.zip › Revised Supplementary Table 1.docx]

**SUPPLEMENTARY TABLE 1** Primer sequences for qPCR assay

| **Primer Name** | **Sequence (5’ to 3’)** |
| --- | --- |
|  |  |
| Mouse P2y1r | Fw CTGACGGTGTTTGCTGTGTCTTAT |
|  | Rev AGAAATAAAGAATGGGGTCCACACA |
| Mouse P2y2r | Fw GGAACCCTGGAATAGCACCATC |
|  | Rev TTCAGGCACAACCCGAGCA |
| Mouse P2y4r | Fw TCTGCCTGAGGAGTTTGACCAC |
|  | Rev GAACGGAGCCGAGAAGATGACT |
| Mouse P2y6r | Fw ATTTCAAGCGACTGCTGCTAACC |
|  | Rev TAGTGAACAGGCATACATCAGGTCC |
| Mouse P2y12r | Fw TTCAGCAGAACCAGGACC |
|  | Rev GTGATGAGCCCAGCAAAG |
| Mouse P2y14r | Fw ATGAACAACTCCACCACCACAGAC |
|  | Rev CAGCCACCACTATGTTCTTGAGATA |
| Mouse Tnf | Fw CCACCACGCTCTTCTGTCTACTG |
|  | Rev GCCATAGAACTGATGAGAGG |
| Mouse IP-10 | Fw ATTGCCCTTGGTCTTCTGA |
|  | Rev CGCACCTCCACATAGCTTAC |
| Mouse Il-6 | Fw GACAAAGCCAGAGTCCTTCAGAGAGATACAG |
|  | Rev TTGGATGGTCTTGGTCCTTAGCCAC |
| Mouse Mcp-1 | Fw TTAAAAACCTGGATCGGAACCAA |
|  | Rev GCATTAGCTTCAGATTTACGGGT |
| Mouse Gfap | Fw TAACGACTATCGCCGCCAACTG |
|  | Rev AGCAAGTGCCTCCTGCTAACTG |
| Mouse Actin | Fw CGTGGGCCGCCCTAGGCACCA |
|  | Rev TTGGCCTTAGGGTTCAGGGGGG |
| Human TNF | Fw CCCCAATCCCTTTATTACCC |
|  | Rev CGAAGTGGTGGTCTTGTTGC |
| Human MCP-1 | Fw CGCCTCCAGCATGAAAGT |
|  | Rev CTTGGATGGTCCATGATAACT |
| Human IP-10 | Fw GCACCATGAATCAAACTG |
|  | Rev ACTAATGCTGATGCAGGTA |
| Human Actin | Fw CATGTACGTTGCTATCCAGGC |
|  | Rev CTCCTTAATGTCACGCACGAT |
