# Supplementary figures and images for: HIV-1 Tat enhances purinergic P2Y4 receptor signaling to mediate inflammatory cytokine production and neuronal damage via PI3K/Akt and ERK MAPK pathways
Source: J Neuroinflammation. 2019 Apr 4;16:71. doi: 10.1186/s12974-019-1466-8 (PMC6449963; doi:10.1186/s12974-019-1466-8)

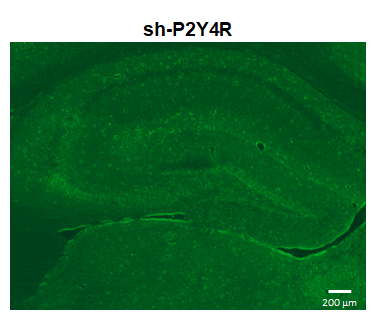

Supplement: Supplementary file 1 — Figure S1. Distribution of GFP in CNS of mice infected by lentivirus. The lentivirus suspension of LV-sh-P2Y4R was injected into mice through the tail vein for 14 days, and then mice were sacrificed and frozen sections (15 μm) from brain tissues. GFP expression was detected under fluorescence microscope (n = 3, original amplification, × 40). Table S1. The list of primer sequences for qPCR assay. (ZIP 263 kb) [file 12974_2019_1466_MOESM1_ESM.zip › Sfig. 1.tif]
